# Familial multiple sclerosis in patients with Von Hippel-Lindau disease

**DOI:** 10.1186/s12883-022-02604-6

**Published:** 2022-03-08

**Authors:** Samir R. Nath, Prabhjot Grewal, Thomas Cho, Yang Mao-Draayer

**Affiliations:** 1 Medical Scientist Training Program, University of Michigan Medical School, Ann Arbor, MI 48109 USA; 2 Department of Neurology, Northwestern University Feinberg School of Medicine, Chicago, IL USA; 3 Department of Neurology, Clinical Autoimmunity Center of Excellence, University of Michigan Medical School, 4015 A. Alfred Taubman Biomedical Sciences Research Building 109 Zina Pitcher Place, Ann Arbor, MI 48109-2200 USA; 4 Graduate Program in Immunology, Program in Biomedical Sciences, University of Michigan Medical School, 4015 A. Alfred Taubman Biomedical Sciences Research Building 109 Zina Pitcher Place, Ann Arbor, MI 48109-2200 USA

**Keywords:** Multiple sclerosis, Von Hippel Lindau, Case report

## Abstract

**Background:**

Multiple sclerosis (MS) is a progressive autoimmune demyelinating disorder. Recent studies suggest that a combination of genetic susceptibility and environmental insult contributes to its pathogenesis. Many candidate genes have been discovered to modulate susceptibility for developing MS by genome wide association studies (GWAS); these include major histocompatibility complex (MHC) genes and non-MHC genes. MS cases in the context of genetic diseases may provide different approaches and clues towards identifying novel genes and pathways involved in MS pathogenesis. Here, we present a case series of two related patients with concomitant Von Hippel-Lindau disease (VHLD) and MS.

**Case presentation:**

We present two patients, a mother (case 1) and daughter (case 2), who developed superimposed relapsing-remitting multiple sclerosis in the background of the autosomal dominant genetic disorder VHLD. Several tumors characteristic of VHLD developed in both cases with pancreatic and renal neoplasms and cerebellar hemangioblastomas. In addition, both patients developed clinical symptoms consistent with multiple sclerosis, supported by radiologic lesions disseminating in time and space.

**Conclusion:**

Though non-MHC susceptibility genes remain elusive in MS, we present the striking finding of superimposed multiple sclerosis in a mother and daughter with VHLD. The VHL gene is known to be the primary regulator of Nrf2, the well-established target of the FDA-approved therapeutic dimethyl fumarate. These cases provide support for further studies to determine whether VHLD pathway related genes represent a novel genetic link in multiple sclerosis.

## Background

Von Hippel-Lindau disease (VHLD) is an autosomal dominant disease characterized by progressive development of a variety of cysts and tumors. These include hemangioblastomas of the CNS and retina, endolymphatic sac tumors, renal cell carcinoma, pheochromocytoma, and pancreatic neuroendocrine tumors, as well as cysts in the kidney, pancreas, and genital tract [1]. Patients are classified as VHLD type 1, typically without pheochromocytoma, and type 2, predominantly with pheochromocytoma [2]. The incidence of VHLD is approximately 1 in 36,000 live births [3, 4], with a penetrance of over 90% by 65 years of age [3], and an average age of onset in the second decade of life [5].

VHLD is caused by deletions or mutations in the VHL gene which encodes for a protein responsible for substrate specificity that is ultimately ubiquitinated [6]. Once ubiquitinated, substrates are bound by the 26S proteasome for degradation [7]. The most well-established targets of the VHL E3 complex are hypoxia-inducible transcription factors (HIF1a and HIF2a) which initiate transcription of many well studied targets, including vascular endothelial growth factor, platelet-derived growth factor B, and erythropoietin. Under normoxic conditions, HIF1a is hydroxylated by prolyl containing hydroxylases, leading to recognition by VBC and subsequent ubiquitination and destruction by the 26S proteasome [8]. However, under hypoxic conditions oxygen is not available for HIF1a hydroxylation, and the HIF1a protein is able to translocate from the cytosol to the nucleus, form a dimer with HIF1B, and initiate transcription of a number of genes involved in growth factor signaling and mitigation of oxidative stress [9].

Deletions or mutations in the VHL gene lead to inability or impaired ability to properly ubiquitinate and subsequently degrade HIF1a. Consequently, increased transcription of HIF1a targets occurs, resulting in increased predilection for development of the various tumors and cysts of VHDL [9]. To our knowledge no other neurological diseases have been associated with VHLD. Multiple sclerosis, on the other hand, is an autoimmune demyelinating disease of the central nervous system with potential genetic predisposition. In this report, we present for the first time a case of superimposed multiple sclerosis in both a mother and daughter with VHLD and highlight the possible connection between the VHL signaling pathway and multiple sclerosis pathophysiology as an exciting area for further study.

## Case presentation

### Case 1

**Fig. 1:**
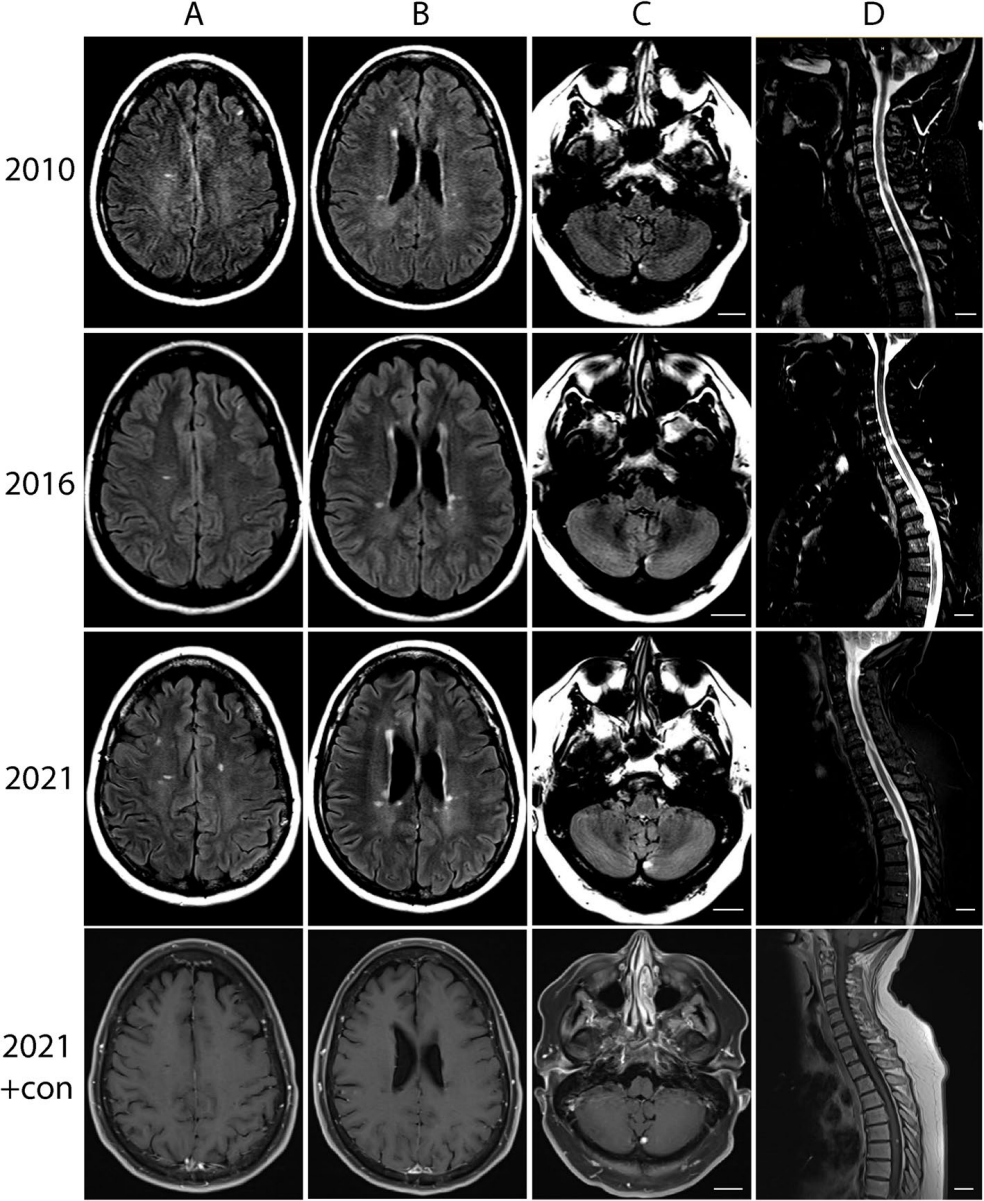
Serial MRI performed in 2010 (row 1), 2016 (row 2), and 2021 (row 3) showing stable cerebellar hemangioblastoma (panel C) and increasing burden of T2 FLAIR hyperintense lesions disseminating in time and space. STIR-weighted image of the spinal cord showing an enhancing lesion in the cervical cord (panel D). Row 4 depicts contrast enhanced T1 sequence of 2021 MRI

A 51-year-old woman was diagnosed with VHLD (C.500G > A, p.Arg167Gln) in 2007 with hemangioblastomas in the spinal cord (T6-T7) and the cerebellum, renal cell carcinoma, pancreatic neoplasm and left ovarian dermoid cyst. She had an episode of vision loss involving the left eye in early 1993 which evolved over a week and recovered over 4–5 months. MRI scan of the brain showed several white matter lesions. She was neurologically asymptomatic until 2010 when she developed left lower extremity weakness and paresthesia following a left partial nephrectomy which resolved over a few months except for subtle weakness of the left hip. She had a repeat episode accompanied by word finding difficulties and increased urinary frequency in 2011. MRI in 2010 and 2011 showed a small hemangioblastoma in the left cerebellum, a T2 hyperintensity in the left C6-C7 cervical cord with no contrast enhancement, and a 4-mm enhancing nodule in T6-T7 consistent with a hemangioblastoma with mild cord edema (Fig. 1, first row). She had mild residual weakness in the left hip and knee flexor muscles.

In 2012, the limb weakness worsened in a waxing and waning pattern with frequent falls and difficulty climbing stairs. She also had decreased vision from the left eye for 2 months, but eye evaluation and visual evoked potentials were normal. Somatosensory evoked potentials showed prolongation of the cortical potential following stimulation of the left posterior tibial nerve suggesting a lesion below C5. Her symptoms spontaneously improved. A diagnosis of MS was raised.

MRI of the brain and spine in January 2016 showed multifocal areas of high signal intensity in the periventricular white matter of both hemispheres. Non-enhancing lesions were also present in the dorsal spinal cord at C2 and C6–7. The hemangioblastoma in the T6-T7 region remained unchanged (Fig. 1, second row). Evaluation showed worsening of left leg weakness with more frequent falls, increased blurry vision in the left eye and declining memory and concentration.

In October 2018, the patient developed increased weakness and burning in both legs, fatigue, and worsened bowel and bladder function. Treatment with corticosteroids showed partial response of symptoms. In February 2020, a new non-enhancing lesion was found in the left frontal lobe on MR. In July, she had decreasing vision in her left eye with color desaturation and pain with eye movement suggestive of optic neuritis which improved after a course of high dose corticosteroids. By October 2020, the patient reported worsening urinary urgency, increased fatigue, and dragging of the left leg and foot. Copaxone was initiated for treating MS. Most recent MRI in February 2021 showed stability of known white matter lesions with no adverse events to date (Fig. 1, third row).

### Case 2

**Fig. 2:**
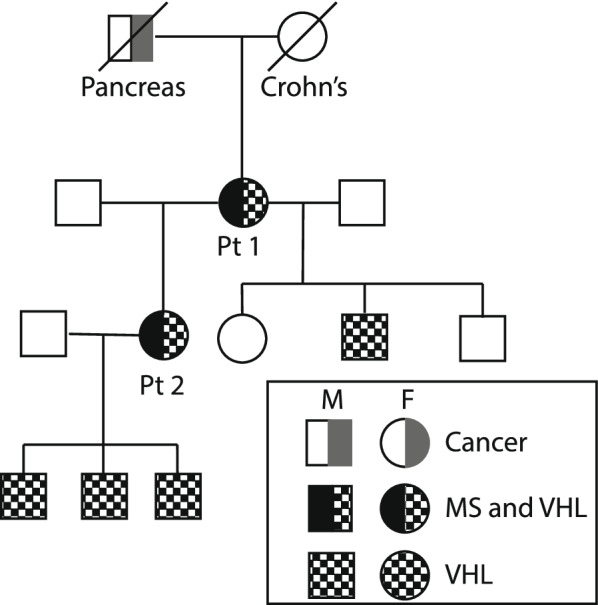
Pedigree of the patients’ family structure. Half-grey symbols: Cancer. Half-black symbols: Multiple Sclerosis. Checkerboard or half-checkerboard symbols: VHLD. Known cancer types and other notable medical conditions are provided as text under each symbol. The patients from each case are labeled as “Pt 1″ and “Pt 2″

The patient’s daughter is a 36-year-old woman diagnosed with VHLD (C.500G > A, p.Arg167Glu) in 2008 with retinal and cerebellar hemangioblastomas, pheochromocytoma, pancreatic neuroendocrine tumor, and recurrent renal cell carcinoma. The patient’s pedigree was significant for multiple cancers in prior generations (Fig. 2). In 2012, surveillance MRI showed few hyperintense subcortical lesions and a prominent C3 lesion without contrast enhancement. MRI in 2019 showed an increase in subcortical and periventricular lesions on T2 weighted images, as well as substantial accumulation of multifocal cervical and thoracic spinal cord lesions.

**Fig. 3:**
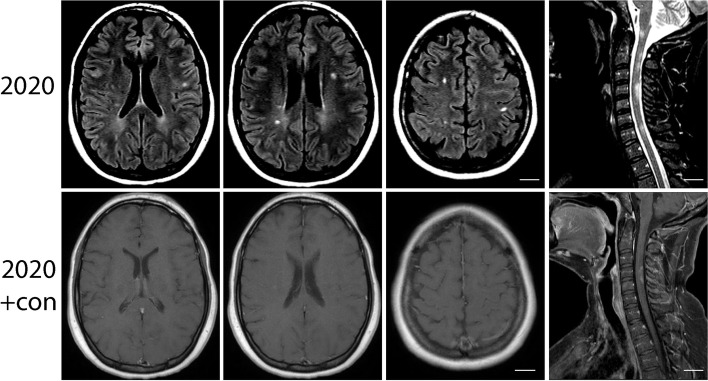
T2-weighted FLAIR MRI of the brain showing subcortical and periventricular lesions in case 2. STIR-weighted image of the spinal cord showing multiple cervical lesions. A subset of these lesions were enhancing (row 2)

In April 2020, she developed an episode of numbness and burning/tingling in her arms and legs for which she was treated with corticosteroids. MRI showed new white matter lesions in the brain and enhancement of the dorsal cervical spine (Fig. 3). Numbness of the hands and feet worsened over the next month. MS mimicker labs including myelin oligodendrocyte glycoprotein or aquaporin 4 antibodies, vitamin B12 and TSH levels were normal. She also developed painful loss of vision in left eye in November 2020, and repeat MRI showed subtle asymmetric contrast enhancement of the left optic nerve consistent with optic neuritis, reinforcing MS diagnosis. Plans were made to initiate Tecfidera and further monitoring is pending follow-up.

## Discussions and conclusions

The mother and daughter cases presented in this study were the first to show concomitant multiple sclerosis in patients with VHLD. Though pure coincidence is possible, it is also possible that mutation of the VHL gene contributed to the development of multiple sclerosis in these cases.

Though the exact pathophysiology of MS is not well characterized, it is thought that a combination of genetic susceptibility and environmental exposure play a role [10]. Many mutations have been identified in histocompatibility antigen (HLA) class I and class II loci which confer increased risk of multiple sclerosis [11–14]. As the HLA system is a fundamental component of antigen presentation and T cell activation, the effect of these alleles is likely increased risk of autoantigen presentation. Several genome wide association studies (GWAS) have identified additional loci which increased susceptibility to multiple sclerosis. Notably, a recent study involving 47,429 multiple sclerosis patients and 68,374 control subjects identified about 200 non-major histocompatibility complex candidate genes [15]. This evidence clearly favors a role for genetic risks in the pathogenesis of the disease.

**Fig. 4:**
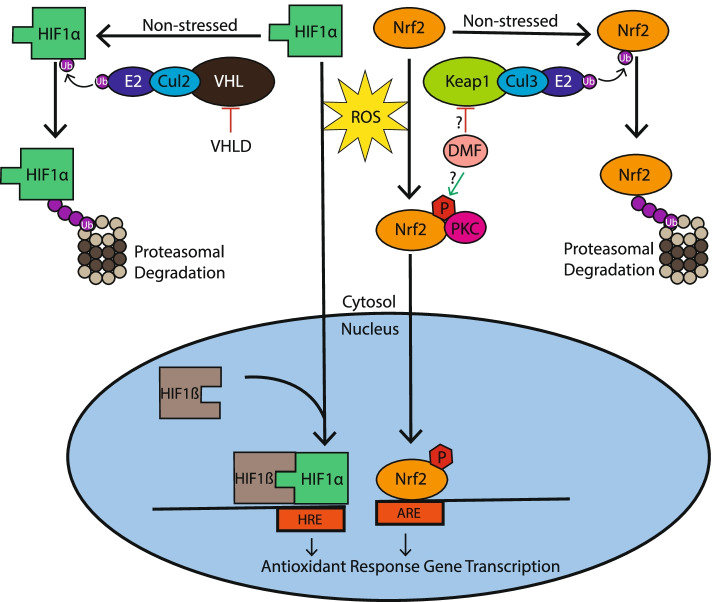
Schematic diagram illustrating the similarities between Nrf2 and HIF1a signaling pathways. *Non-stressed:* HIF1a and Nrf2, under non-stressed conditions, are ubiquitinated and degraded by the proteasome. HIF1a is ubiquitinated by the VHL SCF E3 complex, and Nrf2 is ubiquitinated by the Keap1 SCF E3 complex. *Stressed:* Reactive oxygen species (ROS) are increased, leading to HIF1a translocation and dimerization with HIF1b, binding to Hypoxia Response Elements (HRE) and transcription of antioxidant response genes. ROS also triggers Nrf2 phosphorylation and translocation, binding of Antioxidant Response Elements (ARE), and transcription of antioxidant response genes. *Modulation:* VHLD is driven by inactivating mutation which impairs VHL function. DMF’s proposed mechanisms of action include inhibition of the Keap1 SCF E3 complex and activation of Nrf2 phosphorylation preventing its degradation

The presence of multiple sclerosis in a mother and daughter with VHLD is particularly interesting in light of a recent report of adult oligodendrocyte specific conditional knockout of VHL which showed impaired oligodendrocyte maturation and remyelination in an LPC-induced demyelination mouse model [16]. This direct association between VHL gene deletion and impairment of remyelination may be particularly relevant to pathophysiology of multiple sclerosis subtypes which show oligodendrocyte dystrophy [17]. In addition to this direct association between VHL gene deletion and impairment of remyelination, the downstream targets of VHL have also been implicated in multiple sclerosis pathology. HIF1a, one of the most well-established VHL targets, was recently found to be one of five potential transcriptional regulators of proinflammatory astrocytes in experimental allergic encephalomyelitis mouse models by single cell sequencing [18]. Downstream of the VHL-Hif1a axis, many genes which regulate oxidative stress are expressed. These genes show significant overlap with those of Nrf2, a known therapeutic target in multiple sclerosis (Fig. 4) [19]. Indeed, work from our group has demonstrated that dimethyl fumarate-mediated induction of Nrf2-ERK1/2 MAPK pathway protects neural stem and progenitor stem cells from oxidative stress, leading to decreased stress-induced apoptosis in vitro [20]. Given the overlapping function of Nrf2 and HIF1a in mitigating reactive oxidative species, this represents another mechanism by which the VHL mutation could confer increased susceptibility to multiple sclerosis. These cases provide support for research into determining whether VHL and its related pathway genes represent genetic links to MS and could be potential therapeutic targets in both MS and VHL.

## Data Availability

All data generated or analysed during this study are included in this published article.

## Abbreviations

HIF1a: Hypoxia Inducible Factor 1a
VHLD: Von Hippel-Lindau
SCF: Skp, Cullin, Fbox
ROS: Reactive Oxygen Species
MS: Multiple Sclerosis
Nrf2: Nuclear factor erythroid 2-related factor 2
ARE: Antioxidant Response Element
GWAS: Genome Wide Association Studies
MHC: major histocompatibility complex
TSH: Thyroid Stimulating Hormone
DMF: Dimethyl Fumarate
Ub: Ubiquitin
